# Comparison of biologic hip preservation versus total hip arthroplasty in a preclinical canine model

**DOI:** 10.1093/jhps/hnaf052

**Published:** 2025-09-10

**Authors:** Brent A Prenger, James L Cook, Chantelle C Bozynski, Cristi R Cook, Aaron M Stoker, Keiichi Kuroki, Steven F DeFroda, Brett D Crist

**Affiliations:** Thompson Laboratory for Regenerative Orthopaedics, Hip Preservation Center, Missouri Orthopaedic Institute, University of Missouri, Columbia, United States; Thompson Laboratory for Regenerative Orthopaedics, Hip Preservation Center, Missouri Orthopaedic Institute, University of Missouri, Columbia, United States; Thompson Laboratory for Regenerative Orthopaedics, Hip Preservation Center, Missouri Orthopaedic Institute, University of Missouri, Columbia, United States; Thompson Laboratory for Regenerative Orthopaedics, Hip Preservation Center, Missouri Orthopaedic Institute, University of Missouri, Columbia, United States; Thompson Laboratory for Regenerative Orthopaedics, Hip Preservation Center, Missouri Orthopaedic Institute, University of Missouri, Columbia, United States; Thompson Laboratory for Regenerative Orthopaedics, Hip Preservation Center, Missouri Orthopaedic Institute, University of Missouri, Columbia, United States; Thompson Laboratory for Regenerative Orthopaedics, Hip Preservation Center, Missouri Orthopaedic Institute, University of Missouri, Columbia, United States; Thompson Laboratory for Regenerative Orthopaedics, Hip Preservation Center, Missouri Orthopaedic Institute, University of Missouri, Columbia, United States

## Abstract

While total hip arthroplasty (THA) is an effective treatment option when indicated, young, active individuals are not ideal candidates. Biologic hip preservation (BHP) using femoral head osteochondral and acetabulum labrum allograft transplantation provides a potential alternative. This study was designed to test the hypothesis that BHP would result in superior hip joint function compared to THA in a ‘young adult’ preclinical canine model. With Institutional Animal Care and Use Committee Approval, canine femoral heads and acetabular labrums were aseptically recovered for subsequent transplantation. Ten purpose-bred hounds were randomly assigned to undergo either BHP or THA (*n* = 5 each). Postoperatively, dogs were compared for differences in pain, function, and hip range of motion (ROM), and were evaluated for allograft/implant integration, joint architecture and health, and complications using radiographic, gross, and histologic assessments. At 6 months postoperatively, BHP had significantly (*P* = 0.039) greater hip ROM recovery (97.8% versus 89.9%) and significantly (*P* = 0.02) less pain (0.3 versus 1.4) compared to THA. Radiographic, gross, and histologic assessments supported the safety of BHP. BHP using femoral head osteochondral and acetabulum labrum allograft transplantation was consistently safe and effective in preserving the architecture of the native hip while restoring hip joint health and function with potential advantages over THA for pain relief and hip ROM. Study results support the use of BHP as an alternative option to THA in patients amenable to this treatment strategy. Future studies are needed to delineate the type and extent of hip joint disorders that are amenable to this treatment strategy.

## INTRODUCTION

Traumatic and developmental hip joint disorders, such as fractures, labral tears, avascular necrosis, femoracetabular impingement, and hip dysplasia, can be highly debilitating in young, active individuals and inevitably progress to symptomatic hip osteoarthritis (OA) if not effectively treated [1–7]. Current treatment options for these patients are often limited such that palliative interventions that require activity limitations and lifestyle alterations are common [2–6, 8]. As a result, many patients opt for total hip arthroplasty (THA) once initial interventions have failed to meet their expectations [2–6, 8, 9]. THA is a consistently effective treatment option for end stage OA in older, more sedentary patients. However, young, active individuals are not ideal candidates for THA based on complication and revision rates in conjunction with recommended activity restrictions and lifestyle modifications [2, 3, 6, 9, 10]. Even so, THA is increasingly being used in younger patients in the United States [5, 9, 11]. Importantly, undergoing THA can ‘burn bridges’ in that patients have very limited options for salvage of failed THA procedures. As such, a treatment option that preserves the native hip, restores joint health and function, and allows young, active patients to return to a highly active lifestyle without strict limitations is desirable.

Biologic hip preservation (BHP) strategies have been explored as a potential alternative to THA in select patients [12–18]. Osteochondral allograft transplantation (OCAT) has been the primary option for biologic joint restoration surgeries in the hip. While OCAT has been effective for surgical treatment of large articular defects in other joints, it has been associated with less consistent success in the hip [13–17]. Most hip OCATs have involved isolated focal femoral head defects, and even these are associated with a substantial risk for failure [12, 14, 17, 19]. When acetabular involvement is apparent, risk for failure significantly increases [12–14]. However, recent advances in allograft preservation methods, transplantation techniques, and patient assessment and management strategies have been associated with significant improvements in biologic joint preservation surgery outcomes [14, 20–24]. Implementation of these advances to BHP using optimized femoral head osteochondral and acetabular labrum allograft transplantation has been associated with initial short-term success and potential advantages over THA for the young, active patient population [13–15, 17]. However, direct comparisons between BHP and THA using a head-to-head study design are lacking. Yet, before a head-to-head comparative study can be ethically performed in the clinical setting, additional preclinical evidence is needed to ensure comparative effectiveness safety and value [25–32]. Preclinical canine model studies provide robust translational data for human hip disorders based on the comparative anatomy, biology, biomechanics, and clinically relevant symptoms, diagnostics, and treatment strategies for induced and spontaneously occurring hip pathology [27, 31–33]. Therefore, this study was designed to test the hypothesis that a BHP strategy based on femoral head osteochondral and acetabular labrum allograft transplantation would consistently result in superior hip joint function when compared to THA in a preclinical ‘young adult’ canine model.

## MATERIALS AND METHODS

### Allograft recovery and processing

**Figure 1:**
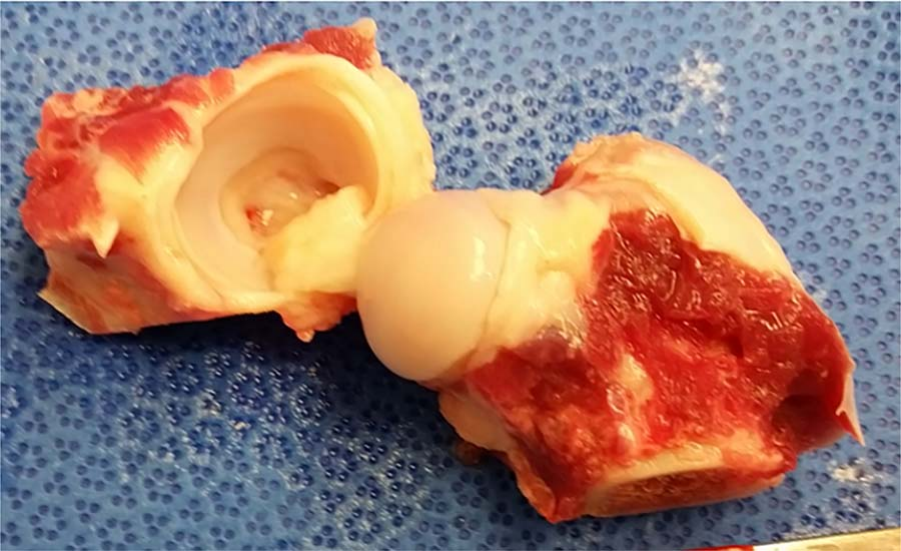
Canine donor femoral head and acetabulum aseptically recovered from a skeletally mature, purpose-bred hound for preservation in the Missouri Osteochondral preservation system (MOPS®, MTF Biologics, Edison, NJ, USA) media for subsequent transplantation.

With Institutional Animal Care and Use Committee (IACUC #9164/9957) approval, femoral heads and acetabula were aseptically recovered from skeletally mature, purpose-bred hounds (*n* = 5: 1–2 years of age, 20.9–31.8 kg) immediately following humane euthanasia performed for reasons unrelated to the present study (Fig. 1). Tissues were thoroughly rinsed with sterile isotonic saline and preserved in individual containers filled with Missouri Osteochondral Preservation System (MOPS®, MTF Biologics, Edison, NJ, USA) media for storage in a clean room at room temperature for 42 days prior to transplantation.

### Surgical procedures

**Figure 2:**
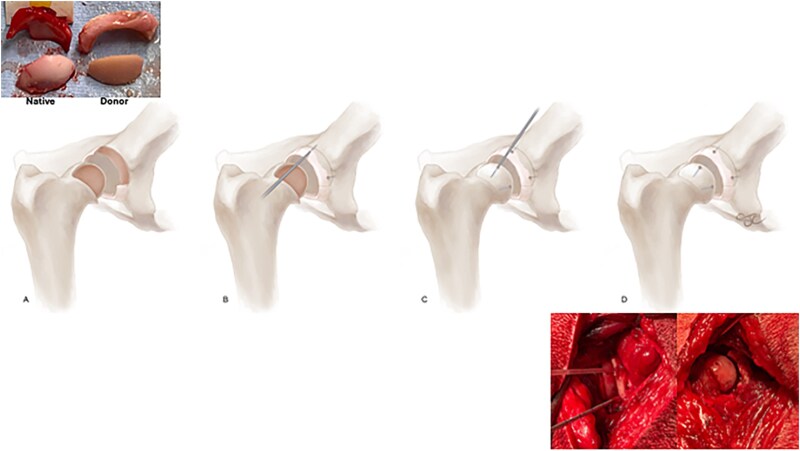
Intraoperative images and illustration of biological hip preservation (BHP) surgery performed in canine hips. The primary load-bearing regions of the acetabular labrum and femoral head were resected and prepared and the defect sizes were measured (A), size-matched, custom-cut donor acetabular labrum-bone-rim and femoral head osteochondral allografts were prepared (top inset) and aseptically transplanted into the prepared beds using bioabsorbable pins (TrimIt, Arthrex, Inc.) for fixation (B, C, D, bottom inset).

**Figure 3:**
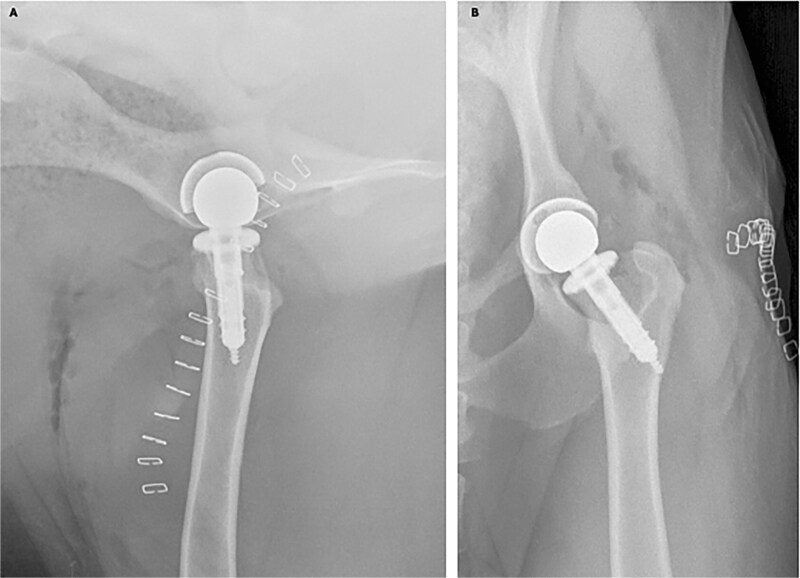
Representative immediate post-operative mediolateral (A) and ventrodorsal (B) radiographic images of size-specific non-cemented total hip arthroplasty performed using canine-specific templating, implants, and instrumentation in canine hips through a craniolateral approach to the hip with deep gluteal partial tenotomy, capsulotomy, and femoral head luxation.

With IACUC approval (IACUC #9167/9961/16680), skeletally mature purpose-bred hounds (n = 10: 1–2 years of age, 22.3–29.7 kg) were premedicated, anaesthetized, and prepared for aseptic surgery of 1 hip. Two doses of cefazolin (22 mg/kg IV) were given 90 minutes apart during the perioperative period. Based on random assignment, the assigned hip in each hound underwent one of the following procedures indicated for symptomatic articular cartilage loss involving the femoral head and acetabulum and not amenable to focal cartilage repair procedures:

- BHP (*n* = 5): Through a craniolateral approach to the hip with deep gluteal partial tenotomy and capsulotomy, the acetabular labrum was first prepared for reconstruction. Concurrent with the surgical approach, bone marrow aspirate (42–60 ml) was obtained from the ipsilateral ilium and processed in the operating room using a commercially available system (Angel, Arthrex, Inc., Naples, FL, USA) to obtain bone marrow aspirate concentrate (BMC), The primary load-bearing region of the acetabular labrum was resected, the defect size was measured, and the recipient bed was decorticated. A size-matched acetabulum was selected from the donor tissues to match the recipient diameter and curvature, and an acetabular labrum-bone-rim allograft was custom cut, prepared and aseptically transplanted into the prepared acetabular bed using two bioabsorbable pins (TrimIt, Arthrex, Inc.) for fixation. Femoral head OCAT was performed using a custom-cut shell allograft prepared from a size-matched femoral head selected from the donor tissues to match the recipient diameter and curvature, to resurface the primary weightbearing portion of the femoral head and aseptically transplanted into the prepared bed using two bioabsorbable pins (TrimIt, Arthrex, Inc.) for fixation. Prior to femoral head OCAT, the recipient bed was prepared by subchondral drilling, and the OCA bone was prepared by subchondral drilling, thorough saline irrigation to remove marrow elements, and saturation with autogenous BMC. Viable cell density (VCD) at time of transplantation was determined, as described below [15, 20–24, 34]. (Fig. 2)
- Total Hip Arthroplasty (THA) (*n* = 5): Through a craniolateral approach to the hip with deep gluteal partial tenotomy and capsulotomy, size-specific non-cemented THA was performed using a canine-specific templating implant and instrumentation system (Arthrex Vet, Naples, FL). Briefly, following capsulotomy, the femoral head was luxated and resected at its junction with the femoral neck. The acetabulum was prepared by reaming to bleeding subchondral bone using the indicated reamer size based on preoperative templating. The corresponding acetabular cup implant was oriented appropriately and impacted into the recipient bed for press-fit fixation. The femur was then prepared by drilling and reaming using the indicated sizes based on preoperative templating. The femoral neck osteotomy surface was prepared by chamfering. The corresponding femoral stem implant was oriented and inserted using screw and press-fit fixation. Trial femoral heads of the appropriate diameter with various neck-length sockets were placed and assessed for stability and hip range-of-motion to allow for selection of the final femoral head implant. The hip was reduced and again assessed for stability and hip range-of-motion (Fig. 3).

Each hip was irrigated with sterile saline and the joint capsule, fascia, subcutaneous tissues, and skin were closed routinely.

### Postoperative management and outcome measures

Postoperatively, dogs were recovered from anaesthesia, monitored daily for general health with assessment and documentation of pain and function, and provided analgesics. For post-operative pain management, a non-steroidal anti-inflammatory analgesic formulated and approved for veterinary uses (carprofen) [4.4 mg/kg SQ] and 2 doses of morphine [0.5 mg/kg IM] were given within 6 hours of the preceding dose initially followed by tramadol [2–7 mg/kg PO] every 12 hours for 3 days and carprofen [4.4 mg/kg PO] once a day for 7 days. Then, throughout the study when VAS pain score (described below) was ≥ 2, carprofen was administered as described above until documented resolution (VAS pain score < 2) [35, 36]. Oral antibiotics (Cefpodoxime 5–10 mg/kg PO SID) were also given for 10 days. The dogs were housed individually and limited to activity in their 25-square-foot runs with enrichment items for six or eight weeks post-operatively, and then allowed daily, fully unrestricted, and monitored out of kennel individual activity, daily monitored group socialization, and leash walking.

All hounds were examined before surgery and at 1, 3, and 6 months (BHP group) or 3 and 12 months (THA group) post-operatively by a board-certified veterinary surgeon blinded to treatment type and time point. Each dog was assessed for general and musculoskeletal health, pain, attitude, appetite, and activity level. Functional outcome measures collected at pre-operative and 3- and 6-month post-operative time points included comfortable hip range of motion (CROM), function score, and pain score. CROM of each hip was measured using a goniometer. With the dog standing, one limb of the goniometer was placed along the lateral axis of the femur and the other arm placed along the lateral axis of the pelvis from the center of the iliac wing to the ischial tuberosity with the hinge point centered over the greater trochanter. The hip was then manually extended to the highest angle the dog tolerated without showing resistance or pain. The extension angle (degrees) noted on the goniometer at this point was recorded. The hip was then manually flexed to the most acute angle the dog tolerated without showing resistance or pain. The flexion angle (degrees) noted on the goniometer at this point was recorded. The flexion angle was subtracted from the extension angle to determine the CROM for each hip. Operated values at each assessment timepoint were compared to pre-operative baseline values in the same hip to determine %CROM for each treated hindlimb: Current CROM/Baseline CROM x 100. Function was evaluated based on visual examination of gait using a 10 cm visual analogue scale (VAS) based on assessment of weightbearing, stance time, stride length, head movement, and load distribution, which has been validated based on strong correlations with pressure mat kinetics [37–40]. Pain was evaluated in each dog by assessing responses associated with pain in dogs (i.e. tensing muscles, resisting, flinching, yelping, turning to look, turning to bite) with the observer recording level of pain using a dynamic (during movement) interactive (associated with palpation of the surgical sites) VAS scale [41]. At the same time points, gait kinetics were quantified by having a dedicated handler trot dogs across a pressure-sensing walkway (GAITFour, Haverton, PA, USA) on-leash at a steady pace with all four footfalls recorded for at least three gait cycles. Using the information gathered from the gait cycles on the pressure-sensing walkway, mean percent body weight distribution of total pressure index (dTPI) was determined for each limb based on total pressure index (TPI). Operated values were compared to unoperated native control hindlimb values to determine %TPI for each treated hindlimb: Operated dTPI/Unoperated dTPI x 100. Dogs were humanely euthanized at the designated timepoint (BHP @ 6 months post-operatively, THA @ 12 months post-operatively) using pentobarbital sodium (Fatal Plus 390 mg/ml, Vortech Pharmaceuticals, Ltd, Dearborn, MI, USA; 1–2 ml/5 kg IV) to allow for endpoint radiographic, gross, and histologic assessments of both hips in each dog.

### Radiographic assessments

Radiographs were interpreted by a board-certified veterinary radiologist to subjectively assess allograft/implant integrity, positioning, and integration, as well as radiographic indicators of hip joint health.

### Gross and histologic assessments

Hips were then disarticulated and processed to allow for gross and histologic assessments of femoral head and acetabulum labrum integrity, integration, and healing. BHP acetabula and femoral heads were collected and fixed in 10% neutral buffered formalin for ~1 week and then decalcified in a solution of 10% ethylenediaminetetraacetic acid (EDTA) until softened (~6 weeks), embedded in paraffin and stained with H&E, Toluidine blue and Picrosirius red. Serial sections of acetabula in the dorsal-ventral orientation were retrieved for plastic and paraffin processing (BHP). THA acetabula and femoral heads were not decalcified and were embedded in methyl methacrylate. Acetabula and associated implants were sectioned in a cranio-caudal orientation. Proximal femurs and associated implants were sectioned longitudinally. THA sections were stained with Stevenel’s blue van Gieson.

Two board-certified veterinary pathologists subjectively evaluated BHP acetabular labrum from each joint for labral graft integrity, integration, and healing based on features described by Domzalski et al. [42]. For THA, subjective histologic assessments of both implants were performed by the same two board certified veterinary pathologists to describe tissue ingrowth (bone and other tissues such as connective tissue), inflammation, and lytic changes.

### Viable cell density

VCD was assessed for unused portions of femoral head OCAs at time of transplantation and transplanted and non-operated femoral heads at time of sacrifice in the BHP cohort. A slow-speed wet diamond saw (Isomet) was used to create ~ 3 mm thick sections of each tissue. The tissue sections were placed in phosphate buffered saline (PBS) containing the live cell stain Calcein AM (1 μg/ml) and the dead cell stain Ethidium Homodimer (2 nM) and incubated for 20 minutes at 37 °C. After incubation, the tissues were rinsed with PBS to remove excess stain and then full-thickness images were obtained at 4X using a fluorescent microscope (Olympus BX51) with attached digital camera (F-View II 12 Bit B&W CCD). To quantify viable chondrocyte density, the number of viable cells in the image stained with Calcein AM was determined using an automated cell counting program (MatLab). The area (mm^2^) of each section counted was measured, and VCD was calculated for each image by dividing the number of viable chondrocytes (vc) counted by the area of the tissue (vc/mm^2^). The VCD of the non-operated femoral head was used as the day-0 VCD for calculating %VCD.

### Statistical analysis

Descriptive data were calculated for each outcome measure. Based on normally distributed (Shapiro–Wilk tests) continuous data, comparisons for statistically significant differences between treatment cohorts were performed using two-tailed unpaired t-Tests. Statistical significance was set at *P* < 0.05.

## RESULTS

All procedures were performed without complications and all hounds survived for the intended study duration with appropriate retention of allografts/implants. Preoperative assessments did not demonstrate any statistically significant differences between BHP and THA cohorts for age, weight, or functional assessments.

### Functional outcomes

By the 3-month post-operative time point, operated hindlimb function mean scores reached > 90% of unoperated controls for both cohorts, which were maintained to study endpoint (Table 1). Similarly, %TPI, representing operated hindlimb loading compared to the unoperated hindlimb, reached > 91% by the 6-month post-operative time point for both cohorts with no statistically significant differences between the two at any assessment timepoint. The difference in %CROM at the 6-month post-operative timepoint was statistical significance (*P* = 0.039) with the BHP cohort having greater return to pre-operative hip ROM compared to the THA cohort. The BHP cohort was also judged to have less pain at each post-operative time point, which reached statistical significance at 6 months post-operatively (*P* = 0.02).

**Table 1:** Mean ± SD longitudinal functional outcome measures for comparisons of biologic hip preservation and Total hip arthroplasty cohorts in a preclinical canine model study

Group	Pain			Function			%TPI		%CROM	
	Pre	3 m	6 m	Pre	3 m	6 m	3 m	6 m	3 m	6 m
BHP	0.2_±0.1_	0.5_±0.5_	0.3_±0.4_	10_±0_	9.1_±0.7_	9.7_±0.6_	89.4_±5.5_	93.2_±9.7_	93.5_±9.6_	97.8_±3.8_
THA	0.2_±0.2_	1.1_±0.7_	1.4_±1_	9.9_±0.3_	9.0_±0.7_	9.8_±0.5_	87.5_±8.3_	91.5_±11.6_	86.8_±12.1_	89.9_±9.4_
*P*-value	0.24	0.15	***0.02***	0.15	0.97	0.75	0.67	0.74	0.11	***0.039***

Key: SD: standard deviation, %TPI: percent total pressure index, %CROM: percent comfortable range of motion, BHP: biologic hip preservation, THA: total hip arthroplasty, Pre: pre-operative, m: month. P-values in bold italic indicate statistically significant (*P* < 0.05) differences between groups

### Radiographic assessments

**Figure 4:**
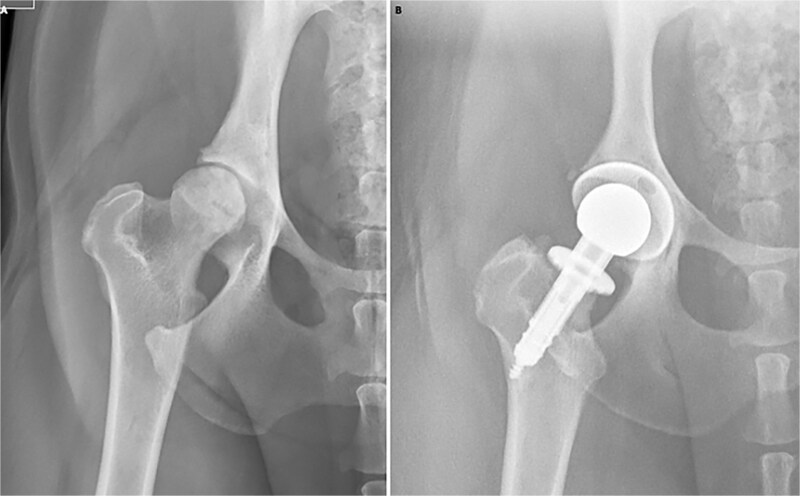
Representative ventrodorsal radiographic images of dogs in BHP (A) and THA (B) cohorts at study endpoint.

Endpoint radiographic imaging assessments documented consistent maintenance of allograft or implant positioning in all dogs in both cohorts (Fig. 4). There were no radiographic signs of allograft rejection, subsidence, articular collapse, fracture, infection, or secondary OA in the BHP cohort. There were no signs of implant subsidence, loosening, fracture, or infection in the THA cohort.

### Viable cell density

**Figure 5:**
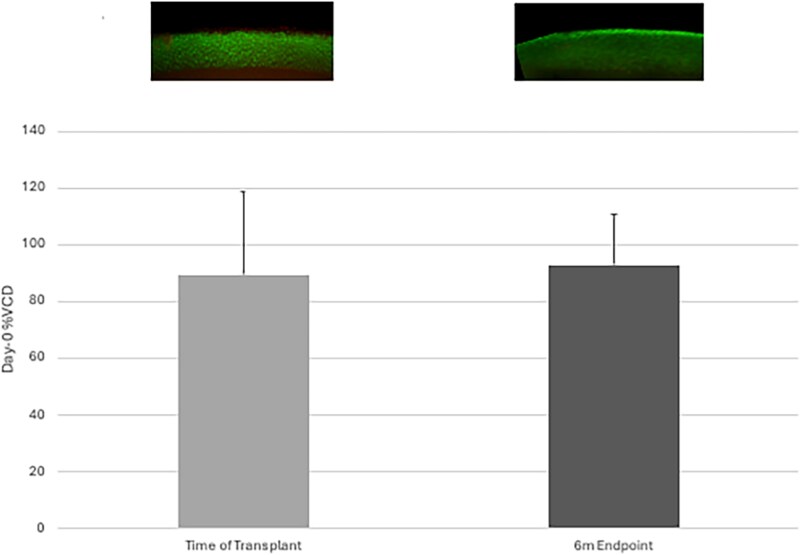
Mean ± standard deviation percentage of Day-0 viable chondrocyte density in femoral heads at time of transplantation and 6-month study endpoint with representative images for each timepoint (Calcein AM-ethidium homodimer live (green)-dead (red) stain, 4x).

Mean %Day-0 VCD of femoral head OCAs was 89.5% at the time of transplantation and 92.8% at the 6-month endpoint (Fig. 5).

### Gross and histologic assessments

**Figure 6:**
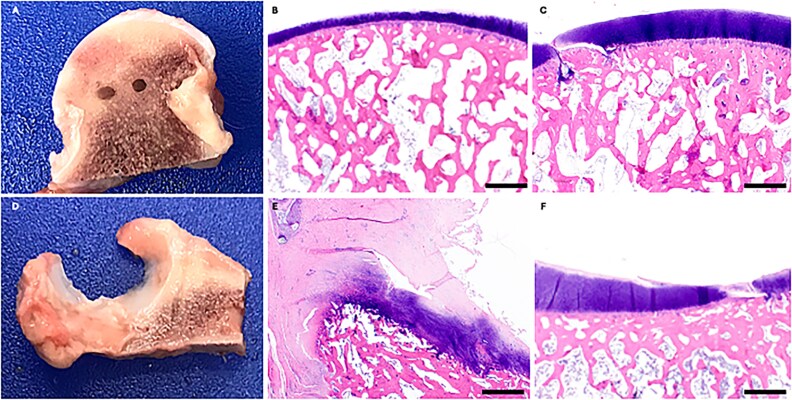
Representative gross images and haematoxylin and eosin-stained photomicrographs of BHP femoral heads (A-C) and acetabulum (D-F) showing consistent maintenance of allograft positioning and integration with native bone, as well as preserved articular tissue architecture. A small gap was consistently present at the articular surface interface between the femoral allograft and native articular cartilage in all specimens as shown in image C. Scale bar = 1 mm.

**Figure 7:**
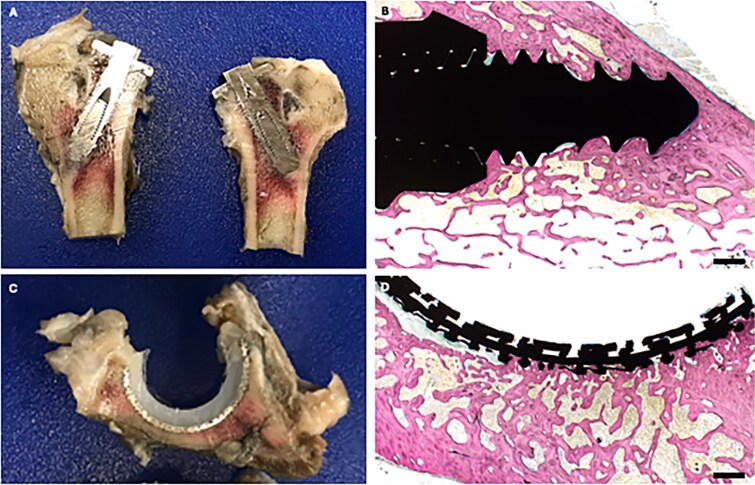
Representative gross images and Stevenel’s blue van Gieson-stained photomicrographs of proximal femur (A-B; with femoral head prosthesis removed at time of tissue processing) and acetabulum (C-D) in the THA cohort. Scale bar = 1 mm.

Endpoint gross and histologic assessments showed consistent maintenance of allograft positioning as well as preserved articular tissue architecture in the BHP cohort **(**Fig. 6**)**. Integration with native bone was complete while a small gap was consistently present at the articular surface interface between femoral head allograft and native articular cartilage in all specimens (Fig. 6C). For the THA implants, consistent robust bony ingrowth was noted for all specimens (Fig. 7).

## DISCUSSION

In this preclinical canine study, BHP using femoral head osteochondral and acetabulum labrum allograft transplantation was consistently safe and effective in preserving the architecture of the native hip while restoring hip joint health and function. Radiographic, gross, and histologic assessments supported the safety of this BHP strategy based on the documented allograft integration, maintenance of hip joint architecture and health, and lack of infection, failure, or joint disease. Functional outcome measures supported the efficacy of this BHP based on highly favourable pain, function, and hip ROM outcomes with statistically significant advantages over THA for pain relief and ROM at 6 months post-operatively.

The highly favourable outcomes noted for the BHP cohort in the present study in comparison to previously reported BHP procedures are likely related to recent advances in allograft preservation methods and transplantation techniques [14, 16, 20–24, 33]. Consistently high viable donor cell density associated with MOPS preservation; pre-implantation subchondral drilling, irrigation, and BMC saturation; and optimized graft preparation and fixation techniques have been associated with significantly improved outcomes after OCAT-based knee biologic joint preservation surgeries [12, 20–24, 26, 31, 33, 43, 44]. In addition, short-term outcomes for combined femoral head and acetabular labrum reconstruction BHP surgeries implementing these advances have also been encouraging [13, 15, 45, 46]. With further optimization based on long-term outcomes and additional preclinical studies, this BHP strategy may emerge as a preferred alternative to THA in a defined patient population. If the safety and sustainable efficacy of combined femoral head and acetabular labrum reconstruction BHP surgeries are validated clinically, the benefits for young, active patients would be substantial. In contrast to THA, successful BHP could allow patients to continue engaging in higher demand occupational, sports, and recreational activities [9–13, 15–17, 21]. Importantly, BHP also addresses the ‘burned bridges’ concern associated with THA in this patient population as it preserves the native hip anatomy such that revision of BHP to THA is associated with outcomes comparable to primary THA in this patient population [9, 12–16, 21]. However, it is important to fully consider the limitations associated with allograft-based BHP, which include allograft access related to donor numbers and regulatory requirements, healthcare insurance-system policies, and costs; allograft availability related to cultural, logistical, and infrastructure limitations; and the need for patient adherence to rigorous recovery and rehabilitation protocols proven essential to functional allograft survival [34, 46, 47].

The current study also has limitations that should be considered when interpreting and applying the results. Preclinical induced-pathology animal model data cannot be expected to completely mimic the clinical scenario, particularly when based on a relatively small sample size and short study duration. However, the canine hip shares many similarities to the human hip regarding anatomy, function, pathology, and treatment strategies, induced-pathology entails more rigorous thresholds for relief of pain and restoration of function, and the outcomes for cohorts in the present study correspond well with those reported for clinical canine patients [25–33, 48]. While the sample size for this study may have limited the post hoc power and potentiated type 2 statistical error, the ethical use of research animals must be the overriding priority for preclinical animal model experimental designs [49]. As such, this study effectively addressed the 3 Rs of ethical research animal use while realizing statistically significant differences in key outcome measures and producing evidence for valid translation to human and veterinary patient populations. Similarly, the 6-month duration for functional assessments with different terminal endpoints for each cohort limits any conclusion regarding the long-term sustainability of the treatment outcomes. Still, the endpoint radiographic, gross, and histologic assessments were designed to evaluate safety of each intervention and not used for comparative effectiveness such that the respective results are valid based on clinical outcomes in canine patients.

Taken together, the results of this study suggest that further preclinical and clinical studies investigating the use of combined femoral head and acetabular labrum reconstruction BHP strategies as an alternative option to THA for the treatment of hip joint disorders in young, active patients are warranted.

## Data Availability

We are willing to provide data related to this study upon request with justification and data use agreement, but we have not contracted with a private or university data hub to share raw data otherwise.

## References

[1] Harris WH . Etiology of osteoarthritis of the hip. Clin Orthop Relat Res 1986;213:20–33. 10.1097/00003086-198612000-00004 3780093

[2] Sankar WN, Nevitt M, Parvizi J et al. Femoroacetabular impingement: defining the condition and its role in the pathophysiology of osteoarthritis. J Am Acad Orthop Surg 2013;21:S7–15. 10.5435/JAAOS-21-07-S7 23818194

[3] Zhang C, Li L, Forster BB et al. Femoroacetabular impingement and osteoarthritis of the hip. Can Fam Physician 2015;61:1055–60. 26668284 PMC4677941

[4] Amanatullah DF, Antkowiak T, Pillay K et al. Femoroacetabular impingement: current concepts in diagnosis and treatment. Orthopedics 2015;38:185–99. 10.3928/01477447-20150305-07 25760499

[5] Gala L, Clohisy JC, Beaulé PE. Hip dysplasia in the young adult. J Bone Joint Surg Am 2016;98:63–73. 10.2106/JBJS.O.00109 26738905

[6] Lespasio MJ, Sodhi N, Mont MA. Osteonecrosis of the hip: a primer. Permanente J 2019;23:18. 10.7812/TPP/18-100 PMC6380478 30939270

[7] Kapron AL, Anderson AE, Peters CL et al. Hip internal rotation is correlated to radiographic findings of cam femoroacetabular impingement in collegiate football players. Arthroscopy 2012;28:1661–70. 10.1016/j.arthro.2012.04.153 22999076

[8] Shi X-T, Li C-F, Han Y et al. Total hip arthroplasty for Crowe type IV hip dysplasia: surgical techniques and postoperative complications. Orthop Surg 2019;11:966–73. 10.1111/os.12576 31755242 PMC6904615

[9] Kurtz SM, Lau E, Ong K et al. Future young patient demand for primary and revision joint replacement: national projections from 2010 to 2030. Clin Orthop Relat Res 2009;467:2606–12. 10.1007/s11999-009-0834-6 19360453 PMC2745453

[10] Malcolm TL, Szubski CR, Nowacki AS et al. Activity levels and functional outcomes of young patients undergoing total hip arthroplasty. Orthopedics 2014;37:e983–92. 10.3928/01477447-20141023-55 25361375

[11] Nho SJ, Kymes SM, Callaghan JJ et al. The burden of hip osteoarthritis in the United States: epidemiologic and economic considerations. J Am Acad Orthop Surg 2013;21:S1–6. 10.5435/JAAOS-21-07-S1 23818185

[12] Baumgartner WT, Shelton TJ, White CR et al. Osteochondral allograft transplantation of the femoral head through an open surgical hip dislocation. J Pediatr Orthop Soc North America 2021;3:287. 10.55275/JPOSNA-2021-287

[13] Oladeji LO, Cook JL, Stannard JP et al. Large fresh osteochondral allografts for the hip: growing the evidence. Hip Int 2018;28:284–90. 10.5301/hipint.5000568 29048690

[14] Kosashvili Y, Raz G, Backstein D et al. Fresh-stored osteochondral allografts for the treatment of femoral head defects: surgical technique and preliminary results. Int Orthop 2013;37:1001–6. 10.1007/s00264-013-1868-7 23553116 PMC3664145

[15] Khanna V, Tushinski DM, Drexler M et al. Cartilage restoration of the hip using fresh osteochondral allograft: resurfacing the potholes. Bone Joint J 2014;96-B:11–6. 10.1302/0301-620X.96B11.34734 25381401

[16] Sherman SL, Garrity J, Bauer K et al. Fresh osteochondral allograft transplantation for the knee: current concepts. J Am Acad Orthop Surg 2014;22:121–33. 10.5435/JAAOS-22-02-121 24486758

[17] Meyers MH . Resurfacing of the femoral head with fresh osteochondral allografts. Long-term results. Clin Orthop Relat Res 1985;111–4. 3893823

[18] Estes BT, Enomoto M, Moutos FT et al. Biological resurfacing in a canine model of hip osteoarthritis. Sci Adv 2021;7:eabi5918. 10.1126/sciadv.abi5918 34524840 PMC8443182

[19] Clark SC, Nagelli CV, DeNovio A et al. Osteochondral allograft and autograft transplant for femoral head defects: a multicenter study. Am J Sports Med 2025;53:1832–40. 10.1177/03635465251338062 40371749

[20] Cook JL, Stoker AM, Stannard JP et al. A novel system improves preservation of osteochondral allografts. Clin Orthop Relat Res 2014;472:3404–14. 10.1007/s11999-014-3773-9 25030100 PMC4182376

[21] Kuroki K, Stoker AM, Stannard JP et al. Biologic joint repair strategies: the Mizzou BioJoint story. Toxicol Pathol 2017;45:931–8. 10.1177/0192623317735786 29020891

[22] Stoker AM, Stannard JP, Cook JL. Chondrocyte viability at time of transplantation for osteochondral allografts preserved by the Missouri Osteochondral preservation system versus standard tissue bank protocol. J Knee Surg 2018;31:772–80. 10.1055/s-0037-1608947 29228404

[23] Cook JL, Stannard JP, Stoker AM et al. Importance of donor chondrocyte viability for osteochondral allografts. Am J Sports Med 2016;44:1260–8. 10.1177/0363546516629434 26920431

[24] Stoker AM, Stannard JP, Kuroki K et al. Validation of the Missouri Osteochondral allograft preservation system for the maintenance of osteochondral allograft quality during prolonged storage. Am J Sports Med 2018;46:58–65. 10.1177/0363546517727516 28937783

[25] Cook JL, Smith PA, Stannard JP et al. A canine hybrid double-bundle model for study of arthroscopic ACL reconstruction. J Orthop Res 2015;33:1171–9. 10.1002/jor.22870 25763560

[26] Cook JL, Hung CT, Kuroki K et al. Animal models of cartilage repair. Bone & Joint Research 2014;3:89–94. 10.1302/2046-3758.34.2000238 24695750 PMC3974069

[27] Pascual-Garrido C, Guilak F, Rai MF et al. Canine hip dysplasia: a natural animal model for human developmental dysplasia of the hip. J Orthop Res 2018;36:1807–17. 10.1002/jor.23828 29227567

[28] Ginja MMD, Ferreira AJ, Jesus SS et al. Comparison of clinical, radiographic, computed tomographic, and magnetic resonance imaging methods for early prediction of canine hip laxity and dysplasia. Vet Radiol Ultrasound 2009;50:135–43. 10.1111/j.1740-8261.2009.01506.x 19400458

[29] Stylianou AP, Guess TM, Cook JL. Development and validation of a multi-body model of the canine stifle joint. Comput Methods Biomech Biomed Engin 2014;17:370–7. 10.1080/10255842.2012.684243 22594487

[30] Pashuck TD, Kuroki K, Cook CR et al. Hyaluronic acid versus saline intra-articular injections for amelioration of chronic knee osteoarthritis: a canine model. J Orthop Res 2016;34:1772–9. 10.1002/jor.23191 26867692

[31] Ewing MA, Stoker AM, Leary EV et al. Treatment-monitoring capabilities of serum and urine biomarkers for meniscal allograft transplantation in a preclinical canine model. Am J Sports Med 2022;50:2714–21. 10.1177/03635465221105481 35834869

[32] Garner BC, Stoker AM, Kuroki K et al. Using animal models in osteoarthritis biomarker research. J Knee Surg 2011;24:251–64. 10.1055/s-0031-1297361 22303754

[33] Crist BD, Stoker AM, Pfeiffer FM et al. Optimising femoral-head osteochondral allograft transplantation in a preclinical model. J Orthop Translat 2016;5:48–56. 10.1016/j.jot.2015.10.001 30035074 PMC5987009

[34] Denbeigh JM, Hevesi M, Paggi CA et al. Modernizing storage conditions for fresh osteochondral allografts by optimizing viability at physiologic temperatures and conditions. Cartilage 2019;13:280S–92. 10.1177/1947603519888798 31777278 PMC8808875

[35] Delgado C, Bentley E, Hetzel S et al. Carprofen provides better post-operative analgesia than tramadol in dogs after enucleation: a randomized, masked clinical trial. J Am Vet Med Assoc 2014;245:1375–81. 10.2460/javma.245.12.1375 25459482 PMC4378264

[36] Southern BL, Long SM, Barnes DN et al. Preliminary evaluation of the effects of grapiprant compared with carprofen on acute pain and inflammation following ovariohysterectomy in dogs. Am J Vet Res 2022;83:1–9. ajvr.21.10.0162. 10.2460/ajvr.21.10.0162 35930789

[37] Besancon MF, Conzemius MG, Derrick TR et al. Comparison of vertical forces in normal greyhounds between force platform and pressure walkway measurement systems. Vet Comp Orthop Traumatol 2003;16:153–7. 10.1055/s-0038-1632766

[38] Lascelles BDX, Roe SC, Smith E et al. Evaluation of a pressure walkway system for measurement of vertical limb forces in clinically normal dogs. Am J Vet Res 2006;67:277–82. 10.2460/ajvr.67.2.277 16454633

[39] Light VA, Steiss JE, Montgomery RD et al. Temporal-spatial gait analysis by use of a portable walkway system in healthy Labrador retrievers at a walk. Am J Vet Res 2010;71:997–1002. 10.2460/ajvr.71.9.997 20807137

[40] Guadalupi M, Crovace AM, Monopoli Forleo D et al. Pressure-sensitive walkway system for evaluation of lameness in dogs affected by unilateral cranial cruciate ligament rupture treated with porous tibial tuberosity advancement. Vet Sci 2023;10:696. 10.3390/vetsci10120696 38133247 PMC10747910

[41] Hudson JT, Slater MR, Taylor L et al. Assessing repeatability and validity of a visual analogue scale questionnaire for use in assessing pain and lameness in dogs. Am J Vet Res 2004;65:1634–43. 10.2460/ajvr.2004.65.1634 15631027

[42] Domzalski ME, Synder M, Karauda A et al. Histological changes of the acetabular labrum in coxarthrosis: labral degeneration and repair. Hip Int 2017;27:66–73. 10.5301/hipint.5000425 27834459

[43] Luk J . Histological evaluation of subchondral bone drilling for preimplantation preparation of osteochondral allografts. Orthopaedic Proceedings 2023;105-B:16. 10.1302/1358-992X.2023.7.016

[44] Baumann CA, Baumann JR, Bozynski CC et al. Comparison of techniques for preimplantation treatment of osteochondral allograft bone. J Knee Surg 2019;32:097–104. 10.1055/s-0038-1636834 29514363

[45] Hevesi M, Spencer-Gardner LS, Krych AJ. Surgical technique: Osteochondral autograft transfer and osteochondral allograft transplant for preservation of the femoral head and acetabulum. In: Nho SJ, Bedi A, Salata MJ et al. (eds.), *Hip Arthroscopy and Hip Joint Preservation Surgery*, pp. 1739–53. Cham: Springer International Publishing, 2022 10.1007/978-3-030-43240-9_101.

[46] Rucinski K, Cook JL, Crecelius CR et al. Outcomes associated with hip preservation using osteochondral allograft transplants and acetabular labrum reconstruction. Hip Int 2025;35:9–17. 10.1177/11207000241288445 39463162

[47] Rucinski K, Cook JL, Crecelius CR et al. Effects of compliance with procedure-specific postoperative rehabilitation protocols on initial outcomes after osteochondral and meniscal allograft transplantation in the knee. Orthop J Sports Med 2019;7:2325967119884291. 10.1177/2325967119884291 31803790 PMC6876180

[48] Franklin SP, Stoker AM, Murphy SM et al. Outcomes associated with osteochondral allograft transplantation in dogs. Front Vet Sci 2021;8:759610. 10.3389/fvets.2021.759610 35004920 PMC8739896

[49] Hubrecht RC, Carter E. The 3Rs and humane experimental technique: implementing change. Animals (Basel) 2019;9:754. 10.3390/ani9100754 31575048 PMC6826930

